# Supplementary material for: Rapid and precise determination of zero-field splittings by terahertz time-domain electron paramagnetic resonance spectroscopy
Source: Chem Sci. 2017 Apr 19;8(11):7312–23. doi: 10.1039/c7sc00830a (PMC5672788; doi:10.1039/c7sc00830a)
Supplement: Supplementary file 1 [file SC-008-C7SC00830A-s001.pdf]

Electronic Supplementary Material (ESI) for Chemical Science.
This journal is © The Royal Society of Chemistry 2017

Rapid and Precise Determination of Zero-Field Splittings by Terahertz Time-Domain Electron Paramagnetic Resonance Spectroscopy

Jian Lu[1], I. Ozge Ozel[2], Carina Belvin[2], Xian Li[1], Grigorii Skorupskii[1], Lei Sun[1], Benjamin K. Ofori-Okai[1], Mircea Dincă[1], Nuh Gedik[2], and Keith A. Nelson[1,*]

[1]*Department of Chemistry, Massachusetts Institute of Technology, Cambridge, Massachusetts 02139, USA*

[2]*Department of Physics, Massachusetts Institute of Technology, Cambridge, Massachusetts 02139, USA*

* kanelson@mit.edu

Supplementary Information

Contents

1. Experimental setup 2
2. Time-domain waveforms and FT amplitude spectra 3
2.1 Hemin 3
2.2 $CoX_2(PPh_3)_2$ 5
2.3 $Fe(H_2O)_6(BF_4)_2$ 6
2.4 $NiCl_2(PPh_3)_2$ 7
3. Spin Hamiltonian 8
4. Determination of *D* and *E* parameters from zero-field and field-dependent EPR measurements 8
5. Materials and pellet preparation 12
6. Powder X-ray diffraction 13
References 14

1. Experimental setup

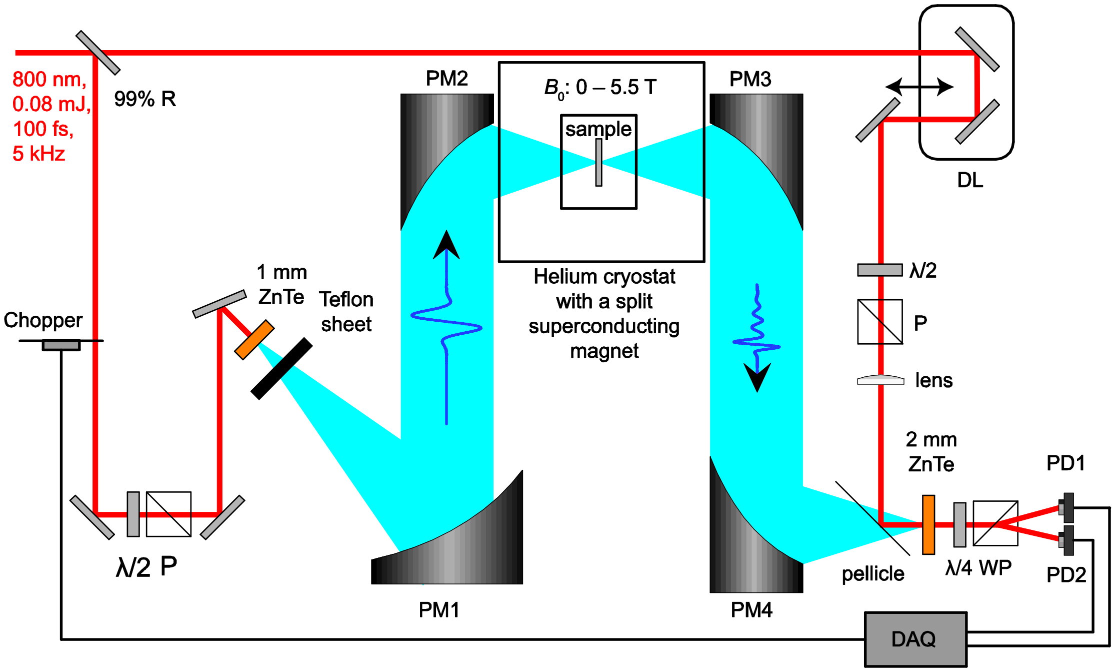

Figure S1. THz time-domain spectroscopy system used in the experiments. DL: delay line; ZnTe: zinc telluride; PM: parabolic mirror; λ/2: half wave-plate; P: polarizer; λ/4: quarter wave-plate; WP: Wollaston prism; PD: photodiode; DAQ: data acquisition card. Red lines indicate optical paths and black lines indicate electrical connections. The blue shaded areas indicate THz beam paths.

Our experimental setup for the tabletop THz time-domain EPR spectroscopy system is shown in basic form in Fig. 1 of the main paper and in more detail schematically in Fig. S1. The laser was a commercial Ti:sapphire amplifier (Spitfire Pro, Spectra Physics) delivering 800 nm pulses with duration of 100 femtoseconds at a repetition rate of 5 kHz. The total output power of 400 mW was split into two optical paths by a 92/8 pellicle beamsplitter. The stronger pulses were modulated at 2.5 kHz by an optical chopper and were incident onto a 1-mm (110)-cut ZnTe crystal to generate THz pulses via optical rectification[1–3]. Single-cycle THz pulses were generated from the ZnTe crystal with a useable bandwidth spanning from 0.1 to 2.5 THz. The residual laser light transmitting through the ZnTe crystal was blocked by a black Teflon sheet. The THz pulses were collimated by a 45-degree off-axis parabolic mirror (PM) and focused onto the sample by a 90-degree off-axis PM (PM1 and PM2, respectively, in Fig. S1). The THz pulses transmitted through the sample and the FID signals that followed them were collimated and focused into a 2-mm ZnTe detection crystal by a pair of 90-degree off-axis PMs (PM3 and PM4 in Fig. S1). The weaker laser pulses from the beamsplitter were time delayed by a delay line (a mechanical translation stage) and attenuated by a half wave-plate (λ/2) and a polarizer (P). They were subsequently focused and overlapped with the THz beam in the ZnTe detection crystal to measure the phase-resolved THz signals via electro-optic sampling[4]. In this measurement, THz electric fields induced a modulation of the refractive indices of the ZnTe crystal along two orthogonal directions. The laser pulses experienced a transient birefringence due to this modulation. The THz-induced birefringence was measured as intensity modulations of the two optical polarization components which were separated by a quarter wave-plate (λ/4) and a Wollaston prism (WP) and detected by a pair of photodiodes (PD1 and PD2). The difference between the measured intensities was detected by a data acquisition card (DAQ) triggered by the chopper. The temporal profiles of the THz pulses and the FID signals were measured with sub-picosecond time resolution by scanning the delay line. The THz beam path was kept under dry air purge, which suppressed THz absorption due to water vapor in the atmosphere. The dynamic range of spectral amplitude of the system was in excess of 10^3 (corresponding to a dynamic range

of spectral intensity in excess of 10^6). Further discussions of the underlying principles of THz generation and detection by optical rectification and electro-optic sampling can be found in References 1-3. The samples were placed in a helium cryostat with a split superconducting magnet (SuperOptiMag, Janis) which could provide static magnetic fields B_0 ranging from 0 to 5.5 T. The orientation of B_0 was perpendicular to the polarization of the THz magnetic field B_1. B_0 can be either parallel or perpendicular to the propagation direction of the THz pulse. The former geometry is called Faraday geometry and the latter is called Voigt geometry[5]. These two geometries do not result in any differences in the EPR measurements. All the elements used in the setup are commercially available.

The time-domain signals measured experimentally typically had 390 time steps of 67 fs. At each time point, the signal was typically averaged for 1000 laser shots. Under these conditions, the data acquisition time for a single absorbance spectrum was roughly 4 minutes, including measuring the time-domain signals for both the reference and the sample. The absorbance spectra reported below were averaged data from 10 measurements, which took roughly 40 minutes to collect. The time window of 26 ps was limited by the THz double reflection in the 1-mm ZnTe crystal used for THz generation. The resulting instrument-limited frequency resolution was approximately 39 GHz. Each spectrum reported here was interpolated by zero padding of the time-domain signals to 4096 data points in the data processing.

In some cases, the oscillatory FID signals emerging from the samples last longer than the instrument-limited time window of 26 ps. The spectra show ringing artifacts due to Fourier transformation of the raw time-domain signals with square windows. A Hamming apodization function was applied to the absorbance spectra, which reduced the ringing effects and had minimal effects on the frequencies of the spin resonance signals of interest.

2. Time-domain waveforms and FT amplitude spectra

2.1 Hemin

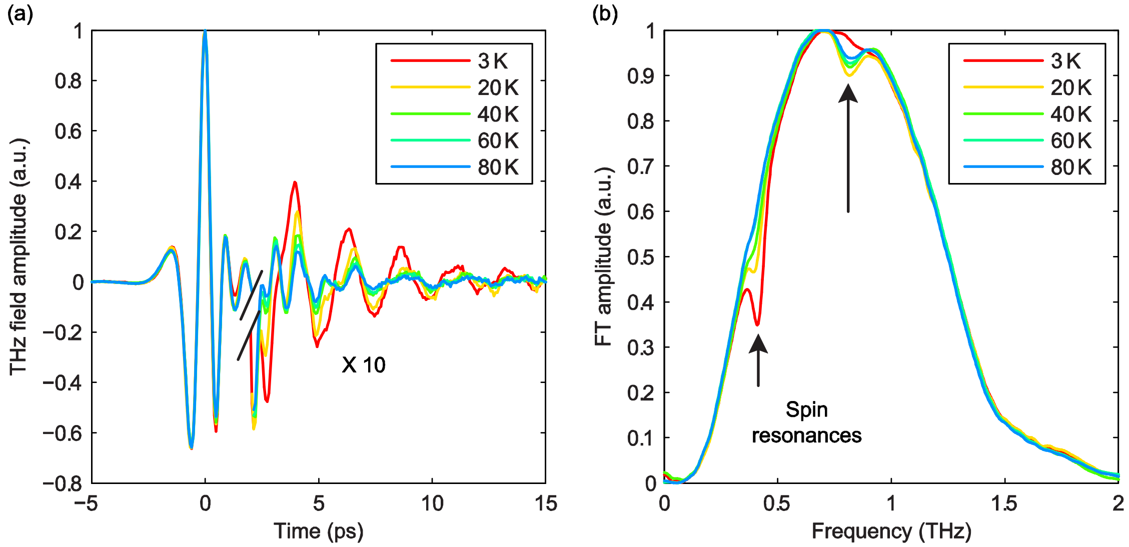

Figure S2. (a) Time-domain waveform of THz pulses transmitted through a pellet of hemin at various temperatures. Oscillatory features following the transmitted THz pulse are identified as the FID signals which are magnified by 10. (b) FT amplitude spectra of the THz pulses transmitted through hemin at various temperatures obtained by numerical Fourier transformation of time-domain waveforms in (a). The spin resonances are indicated by the arrows. The data are color-coded according to the temperatures shown in the legends.

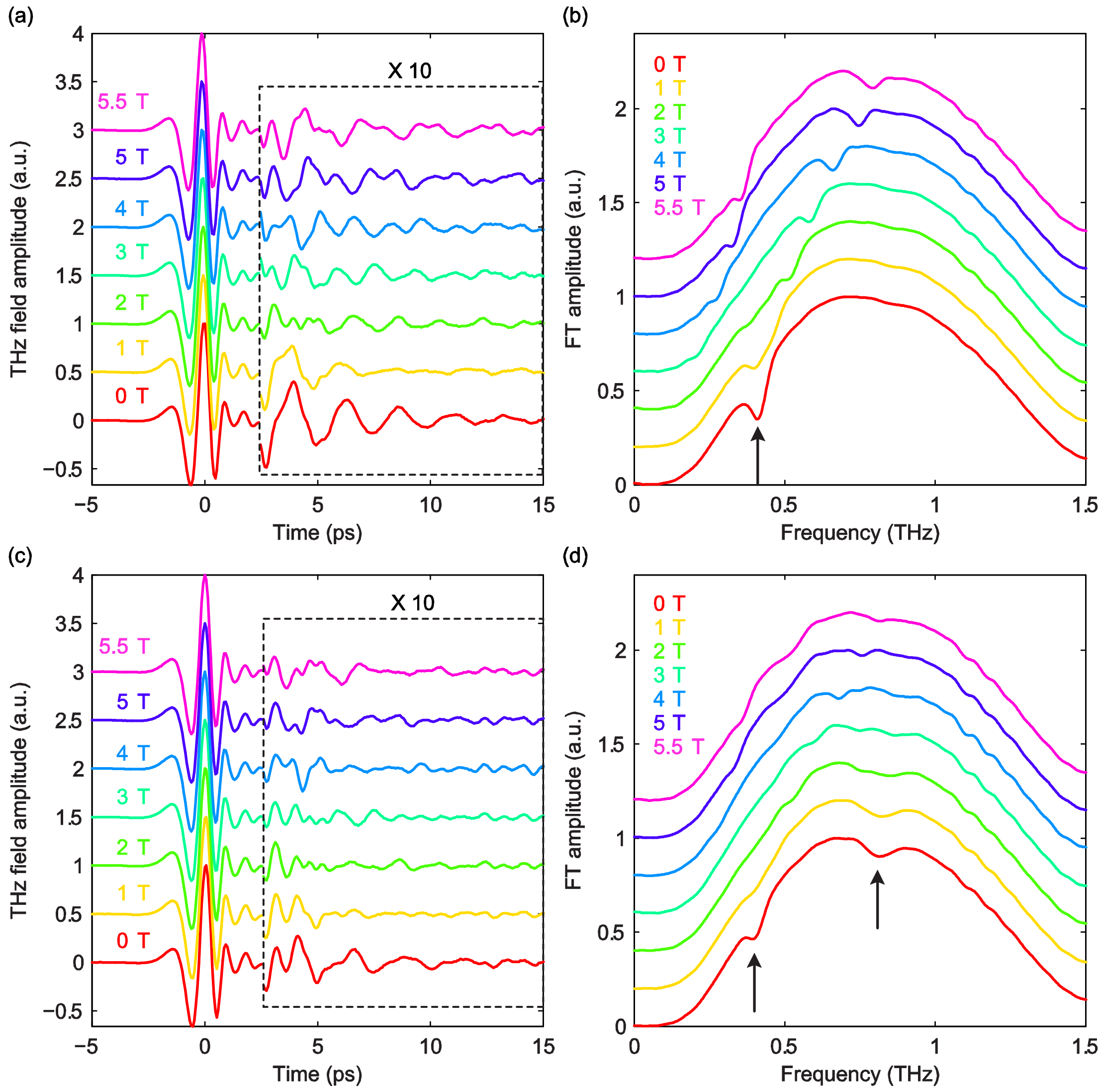

Figure S3. (a) Time-domain FID traces and (b) FT amplitude spectra as functions of B_0 for hemin at 3 K. (c) Time-domain FID traces, and (d) FT amplitude spectra as functions of B_0 for hemin at 20 K. The time-domain traces and spectra are color-coded according to the values of B_0 indicated. In (a) and (c), the FID signals in the dashed boxes are magnified by 10 to bring out the weak signals. In (c) and (d), the arrows indicate the spin resonances at zero field.

2.2 $CoX_2(PPh_3)_2$

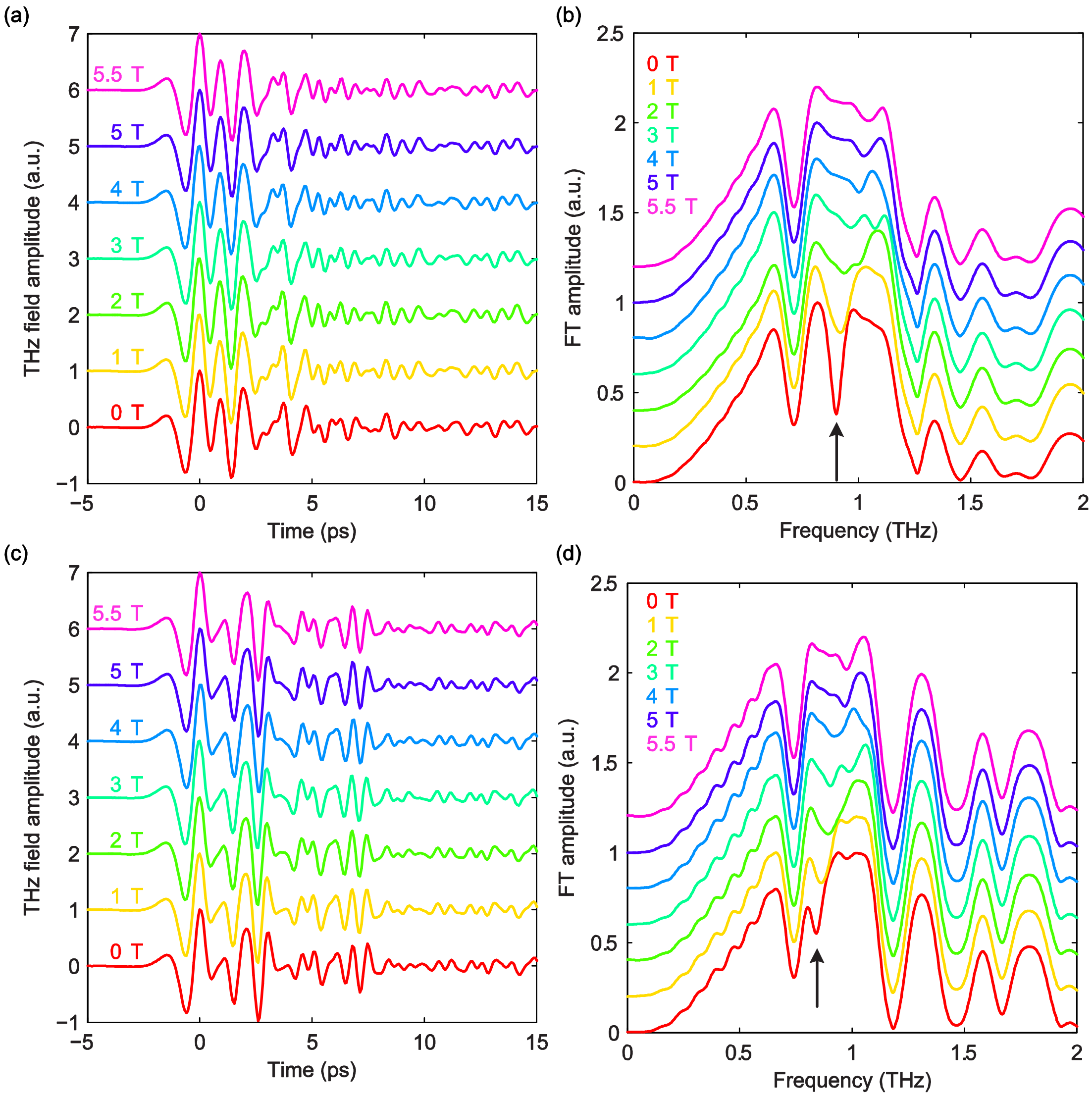

Figure S4. (a) Time-domain FID traces and (b) FT amplitude spectra as functions of B_0 for $CoCl_2(PPh_3)_2$ at 6 K. (c) Time-domain FID traces, and (d) FT amplitude spectra as functions of B_0 for $CoCl_2(PPh_3)_2$ at 2 K. The time-domain traces and spectra are color-coded according to the values of B_0 indicated. In (a) and (c), the signals are dominated by vibrational FIDs. In (c) and (d), a Hamming apodization function was applied to each spectrum. The arrows indicate the spin resonances at zero field.

2.3 $Fe(H_2O)_6(BF_4)_2$

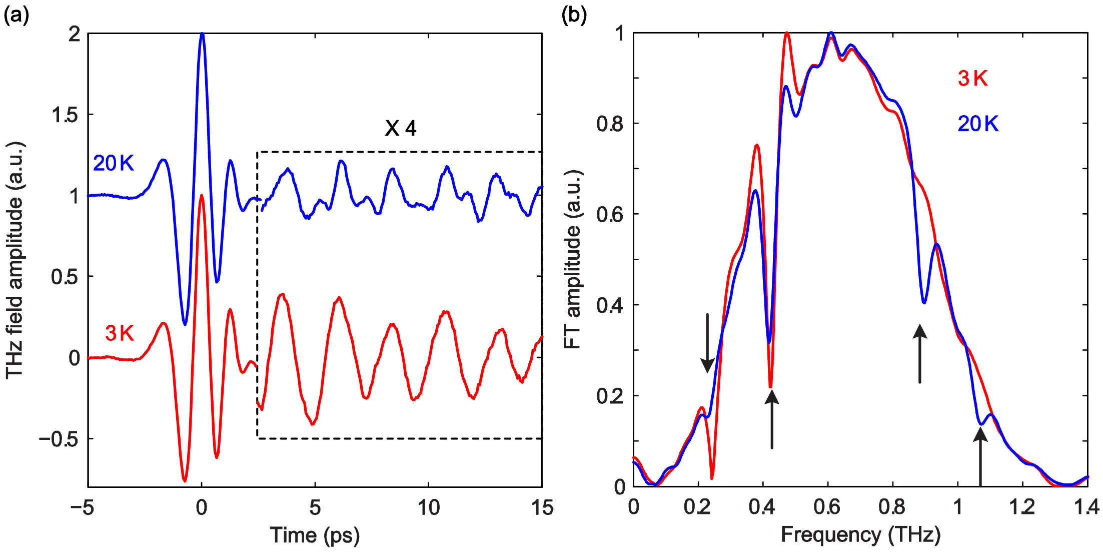

Figure S5. (a) Time-domain FID traces and (b) FT amplitude spectra for $Fe(H_2O)_6(BF_4)_2$ at 1.8 K (red) and 20 K (blue). The traces and spectra are color-coded according to the temperatures indicated. In (a), the FID signals are magnified by 4. In (b), a Hamming apodization function was applied to each spectrum. The arrows indicate the spin resonances at zero field.

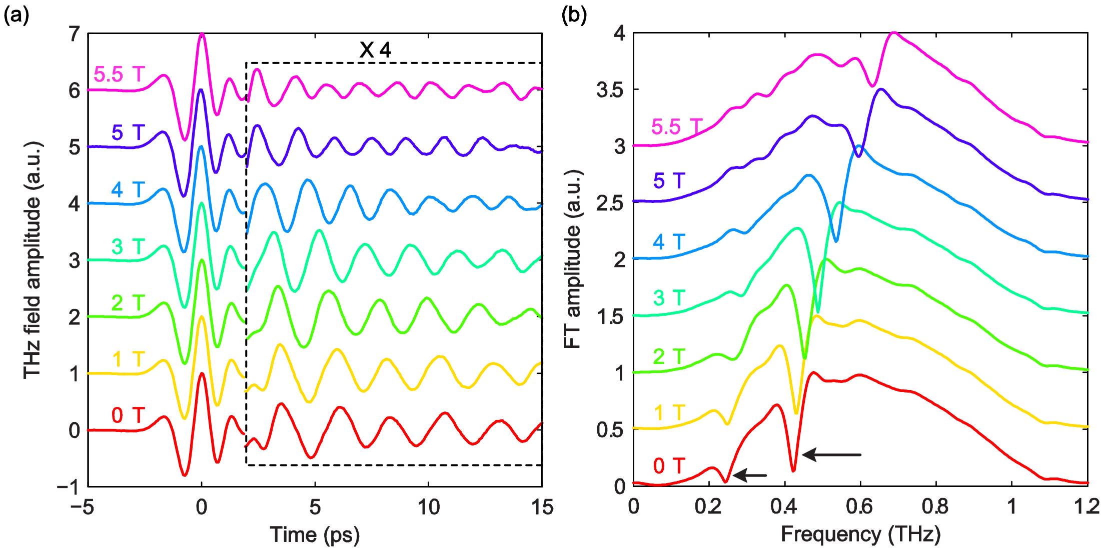

Figure S6. (a) Time-domain FID traces and (b) FT amplitude spectra as functions of B_0 for $Fe(H_2O)_6(BF_4)_2$ at 1.8 K. The traces and spectra are color-coded according to the values of B_0 indicated. In (a), the FID signals in the dashed box are magnified by 4. In (b), a Hamming apodization function was applied to each spectrum.

2.4 $NiCl_2(PPh_3)_2$

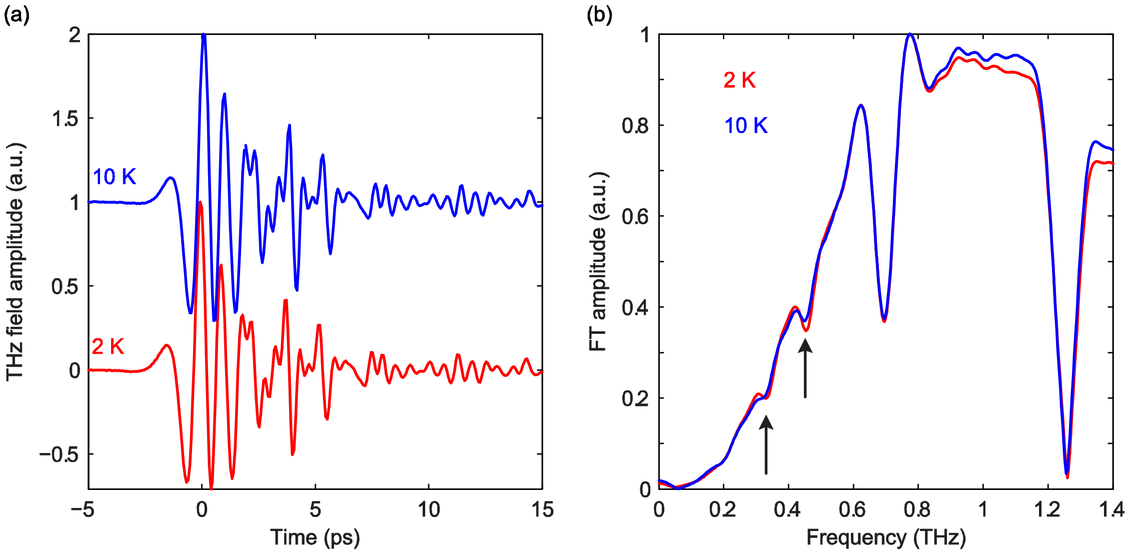

Figure S7. (a) Time-domain FID traces and (b) FT amplitude spectra for $NiCl_2(PPh_3)_2$ at 2 K (red) and 10 K (blue). The traces and spectra are color-coded according to temperatures indicated. In (a), the signals are dominated by vibrational FIDs. In (b), a Hamming apodization function was applied to each spectrum. The arrows indicate the spin resonances at zero field.

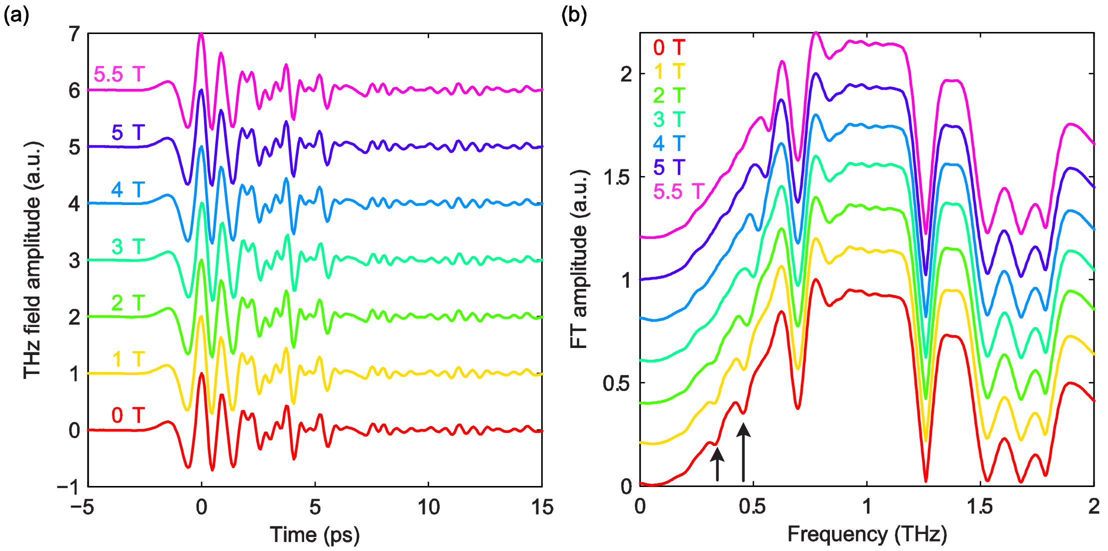

Figure S8. (a) Time-domain FID traces and (b) FT amplitude spectra as functions of B_0 for $NiCl_2(PPh_3)_2$ at 2 K. The time-domain traces and spectra are color-coded according to the values of B_0 indicated. In (a), the FID signals are dominated by vibrational signals. In (b), a Hamming apodization function was applied to each spectrum. The arrows indicate the spin resonances at zero field.

3. Spin Hamiltonian

The spin Hamiltonian[6,7] discussed in the main text consists of the ZFS and EZI terms. The general form of the ZFS parameter $\bar{\bar{D}}$ is a second-rank tensor, which is set traceless (the sum of the diagonal components is zero) and symmetric ($D_{ij} = D_{ji}$). The general form of the spin Hamiltonian describing the ZFS for a single spin system is written as

$$\hat{H}_{ZFS} = \hat{S} \cdot \bar{\bar{D}} \cdot \hat{S}_0^T, \ (S1)$$

where $\hat{S} = [\hat{S}_x \quad \hat{S}_y \quad \hat{S}_z]$ is the spin vector, and $\hat{S}_i \ (i = x,y,z)$ are the spin matrices. In the eigenframe where the D tensor is diagonal, the ZFS Hamiltonian can be transformed into the commonly written form as,

$$\hat{H}_{ZFS} = D\left[\hat{S}_z^2 - \frac{1}{3}S(S+1)\right] + E\left(\hat{S}_x^2 - \hat{S}_y^2\right), \ (S2)$$

where S is the total spin quantum number, and D and E are the axial and transverse ZFS parameters, given by[7] $D = \frac{3}{2}D_{zz}$ and $E = \frac{D_{xx} - D_{yy}}{2}$. For a spin-$S$ system, the spin matrices are square matrices of dimension $2S + 1$. They are always represented in the Zeeman basis with states $|S,M_S\rangle$ ($M_S = -S, -S+1,...,S$). The states are usually denoted by $|M_S\rangle$ for short. The spin matrices can be constructed using the following relations

$$\langle M_S^{'} | \hat{S}_x | M_S \rangle = \left(\delta_{M_S^{'},M_S+1} + \delta_{M_S^{'}+1,M_S}\right)\frac{1}{2}\sqrt{S(S+1) - M_S^{'}M_S}, \ (S3a)$$

$$\langle M_S^{'} | \hat{S}_y | M_S \rangle = \left(\delta_{M_S^{'},M_S+1} - \delta_{M_S^{'}+1,M_S}\right)\frac{1}{2i}\sqrt{S(S+1) - M_S^{'}M_S}, \ (S3b)$$

$$\langle M_S^{'} | \hat{S}_x | M_S \rangle = \delta_{M_S^{'},M_S} M_S, \ (S3c)$$

where δ is the Kronecker delta. The ZFS energy levels of the spin systems studied in the main text can be derived from these equations.

The general form of the EZI term is written in the tensor form as

$$\hat{H}_{EZI} = \mu_B \hat{S} \cdot \bar{\bar{g}} \cdot B_0^T, \ (S4)$$

where μ_B is Bohr magneton and $B_0 = [B_{0x} \quad B_{0y} \quad B_{0z}]$ is the applied static magnetic field vector, and $\bar{\bar{g}}$ is the g-factor, which is a tensor. The g-factor is usually symmetric and can be transformed into diagonal form, with the diagonal elements $g_i(i = x,y,z)$ determined from experimental measurements.

4. Determination of *D* and *E* parameters from zero-field and field-dependent EPR measurements

As shown in the main paper, zero-field EPR measurements yield the absolute values $|D|$ and $|E|$ of the ZFS parameters for integer spin systems, in which the spin sublevels are nondegenerate. For $S = 3/2$ systems, degeneracies among the sublevels limit the zero-field EPR measurements to determination of combinations of the ZFS parameters, as D and E cannot be separately determined from one doubly degenerate transition at $\sqrt{D^2 + 3E^2}$ measured in zero-field EPR. Application of an external magnetic field shifts the levels, enabling separate determination of the absolute values $|D|$ and $|E|$ for $S = 3/2$ systems. For other half-integer spin

systems, zero-field EPR can measure more than one (doubly degenerate) magnetic dipole-allowed transition derived from the magnetic sublevels, so $|D|$ and $|E|$ can be determined without application of an external field.

Variation of the temperature in the presence of a magnetic field allows determination of the sign of D for $S = 3/2$ systems and for $S = 1$ systems with zero E parameter values. In all other spin systems, variation of the temperature at zero magnetic field is sufficient for determination of the sign of D. The details with examples of $S = 3/2$, $S = 5/2$, $S = 1$ and $S = 2$ systems are elaborated briefly below.

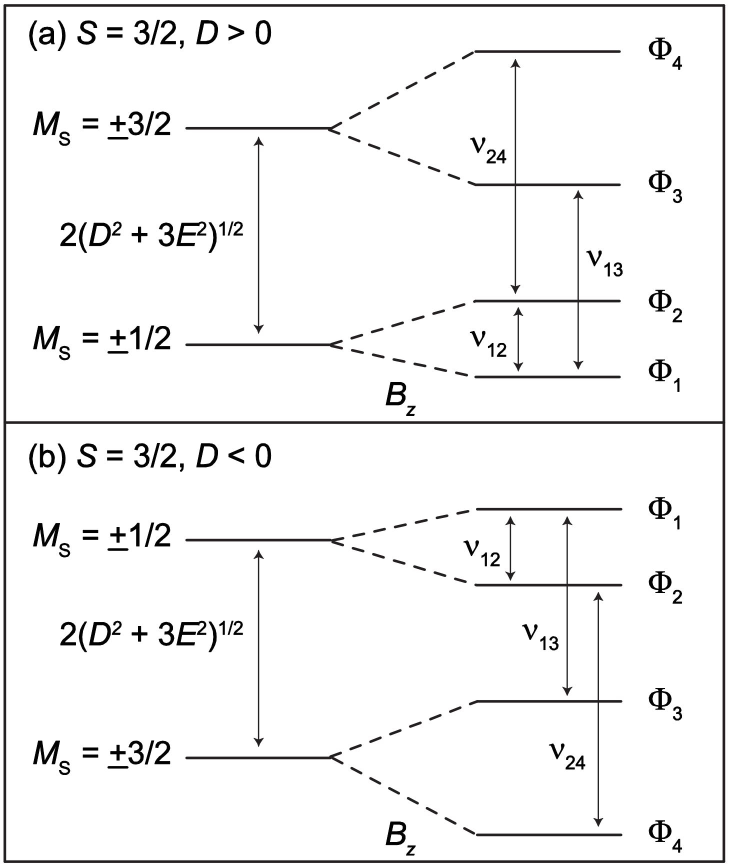

Figure S9. Magnetic sublevel energy diagrams for $S = 3/2$ systems with a positive D (a) or a negative D (b). Though the magnetic sublevels are labeled with M_S, note that M_S is a "good" quantum number only for $E = 0$. In each case, applying an external magnetic field along the molecular z-axis, the Kramers doublets are split which allows determination of the sign of D.

In order to determine the sign of the D parameter in $S = 3/2$ systems, EPR spectra with varying external magnetic field and temperature are necessary. As an example, we show in Fig. S9 the magnetic sublevel energy diagrams for $S = 3/2$ systems with positive or negative D. Without splitting the Kramers doublets, the EPR transition frequencies are both $\sqrt{D^2 + 3E^2}$. We assume an external magnetic field B_z along the molecular z-axis, which can be achieved with a single-crystal sample. (For powder samples, the molecular orientation is random with respect to the magnetic field. Zeeman interactions with an anisotropic g tensor result in somewhat complex lineshapes and need to be analyzed numerically. ZFS and g tensor parameters can be obtained with high precision from field-swept EPR measurements combined with spin Hamiltonian simulations[5,8].) The applied field splits the Kramers doublets, separating the two transitions that are overlapped in the zero-field spectrum and also introducing a new magnetic dipole-allowed transition at frequency ν_{12} between the Φ_1 and Φ_2 levels (derived from $M_S = \pm 1/2$ states that were degenerate under zero field). The spectral amplitude of the new peak exhibits different trends for different signs of D as the

temperature is varied, due to the Boltzmann factor. For positive *D*, states Φ_1 and Φ_2 have lower energies than states Φ_3 and Φ_4 and the ν_{12} transition has an increasing spectral amplitude as temperature is reduced and population is increased in Φ_1. For negative *D*, states Φ_1 and Φ_2 have the higher energies and the ν_{12} transition has a decreasing spectral amplitude as temperature decreases. The external magnetic field also allows separate determination of *D* and *E*. The frequencies of the magnetic dipole-allowed transitions shown in Fig. S9 are analytically given by

$$\nu_{12} = g_z\mu_B B_z + \left(D^2 + 3E^2 - 2Dg_z\mu_B B_z + g_z^2\mu_B^2 B_z^2\right)^{\frac{1}{2}} - \left(D^2 + 3E^2 + 2Dg_z\mu_B B_z + g_z^2\mu_B^2 B_z^2\right)^{\frac{1}{2}}; \qquad (S5a)$$

$$\nu_{13} = 2\left(D^2 + 3E^2 - 2Dg_z\mu_B B_z + g_z^2\mu_B^2 B_z^2\right)^{\frac{1}{2}}; \qquad (S5b)$$

$$\nu_{24} = 2\left(D^2 + 3E^2 + 2Dg_z\mu_B B_z + g_z^2\mu_B^2 B_z^2\right)^{\frac{1}{2}}. \qquad (S5c)$$

By analyzing the field-dependences of these three transition frequencies from field-swept EPR spectra, we can obtain the g_z factor and the *D* and *E* parameters.

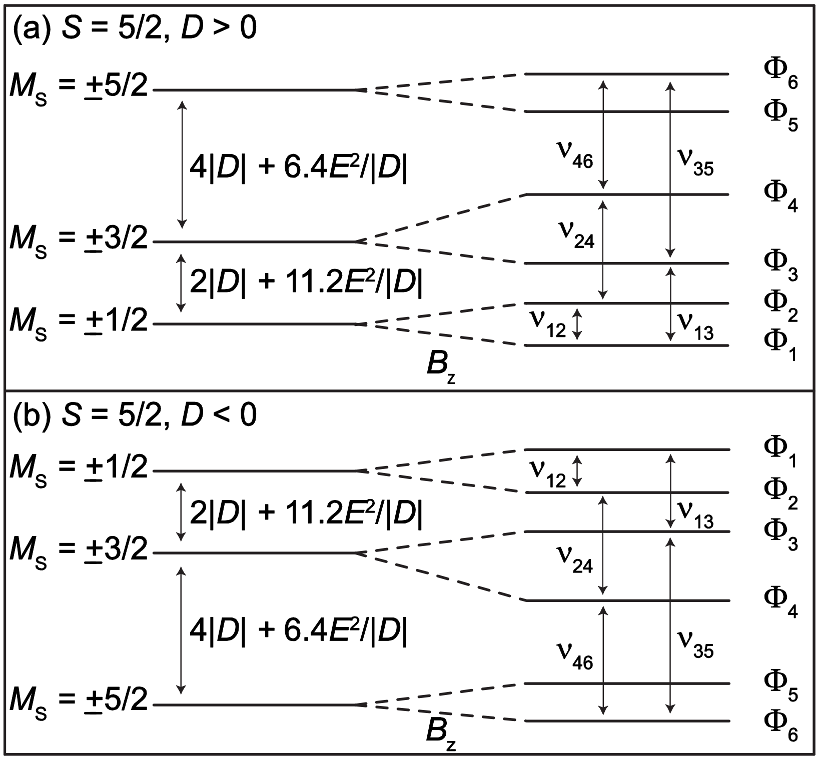

Figure S10. Magnetic sublevel energy diagrams for $S = 5/2$ systems with (a) positive or (b) negative *D*. Though the magnetic sublevels are labeled with M_S, note that M_S is a "good" quantum number only for $E = 0$. The transition frequencies are calculated through second-order perturbation theory. *D* and *E* can both be determined from the two transition frequencies measured at zero field. The sign of *D* can be determined through temperature-dependent measurements at zero field.

In half-integer spin systems other than $S = 3/2$, the degeneracies of magnetic dipole-allowed transitions are partially removed by nonzero *D*, which allows separate determination of $|D|$ and $|E|$ and determination of the sign of *D* at zero field. The magnetic sublevel energy diagrams for $S = 5/2$ systems are shown in Fig. S10. Zero-field EPR measurements at a fixed temperature measure the magnetic dipole-allowed transitions

between $M_S = \pm 1/2$ and $M_S = \pm 3/2$ and between $M_S = \pm 3/2$ and $M_S = \pm 5/2$, whose frequencies are calculated by second-order perturbation theory[9] to be $2|D| + 11.2E^2/|D|$ and $4|D| + 11.2E^2/|D|$ in both cases. Hence for spin-5/2 systems, D and E can be separately determined by measuring these two frequencies at zero external magnetic field. The sign of D can be deduced from zero-field EPR spectra with varying temperature. If D is positive, $M_S = \pm 1/2$ states are lowest in energy. As temperature decreases, the lower-frequency transition amplitudes between $M_S = \pm 1/2$ and $M_S = \pm 3/2$ states increase monotonically while the higher-frequency transition amplitude between $M_S = \pm 3/2$ and $M_S = \pm 5/2$ states increases first and then decreases as the thermal population in states $M_S = \pm 3/2$ decreases. If D is negative, $M_S = \pm 5/2$ states are lowest in energy. As temperature decreases, the higher-frequency transition amplitude between $M_S = \pm 5/2$ and $M_S = \pm 3/2$ states increases monotonically while the lower-frequency transition amplitude between $M_S = \pm 3/2$ and $M_S = \pm 1/2$ states increases first and then decreases as the thermal population in states $M_S = \pm 3/2$ decreases. Applying an external magnetic field further splits the degenerate doublets, allowing determination of the *g*-factor through field-dependent EPR measurements.

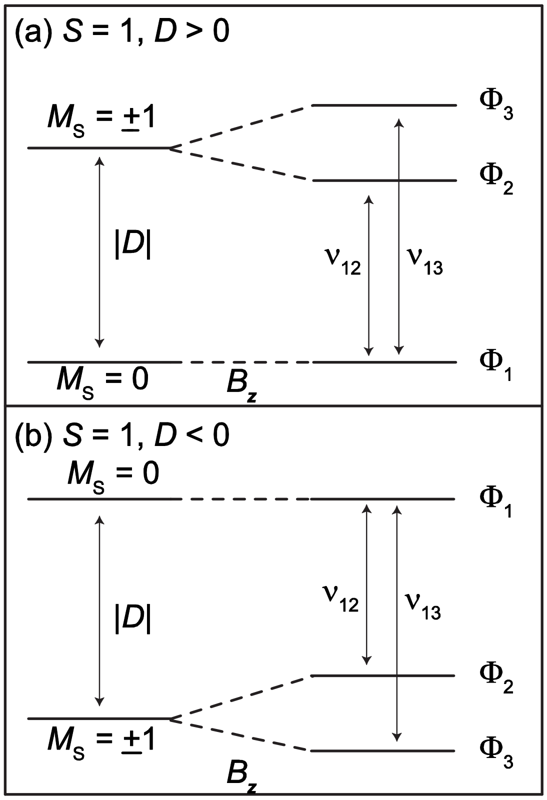

Figure S11. Magnetic sublevel energy diagrams for $S = 1$ systems with $E = 0$ and (a) positive or (b) negative D. Applying an external magnetic field B_z along the molecular z-axis, the $M_S = \pm 1$ doublet is split, which allows determination of the sign of D.

For $S = 1$ systems with a zero (or nonzero) E parameter, zero-field EPR measurement yields $|D|$ in both cases. An external magnetic field is required to split the doublet with $M_S = \pm 1$ as shown in Fig. S11. The sign of D can then be determined by the temperature-dependent changes in spectral amplitudes of the magnetic dipole-allowed transitions. As temperature decreases, the spectral amplitudes at ν_{12} and ν_{13} both increase monotonically if D is positive. If D is negative, the higher-frequency transition amplitude at ν_{13}

increases monotonically while the lower-frequency transition amplitude at ν_{12} increases first and then decreases as the thermal population in state Φ_2 decreases. Nonzero *E* values also can be determined through zero-field EPR measurements as illustrated in the main paper.

The magnetic sublevel energy diagrams for *S* = 2 systems are shown in Fig. S12. With *E* = 0, zero-field EPR measurements at a fixed temperature measure the magnetic dipole-allowed transitions between $M_S = 0$ and $M_S = \pm 1$ and between $M_S = \pm 1$ and $M_S = \pm 2$, whose frequencies are $|D|$ and $3|D|$ respectively regardless of the sign of *D*. As temperature decreases, the lower-frequency transition amplitudes between $M_S = 0$ and $M_S = \pm 1$ states increase monotonically while the higher-frequency transition amplitude between $M_S = \pm 1$ and $M_S = \pm 2$ states increases first and then decreases as the thermal population in states $M_S = \pm 1$ decreases if *D* is positive. If *D* is negative, as temperature decreases, the higher-frequency transition amplitude between $M_S = \pm 1$ and $M_S = \pm 2$ states increases monotonically while the lower-frequency transition amplitude between $M_S = 0$ and $M_S = \pm 1$ states increases first and then decreases as the thermal population in states $M_S = \pm 1$ decreases. With nonzero *E*, all the degeneracy is removed as shown in the main paper. Once again, based on different trends of the spectral amplitudes as functions of temperature one can determine the sign of *D*.

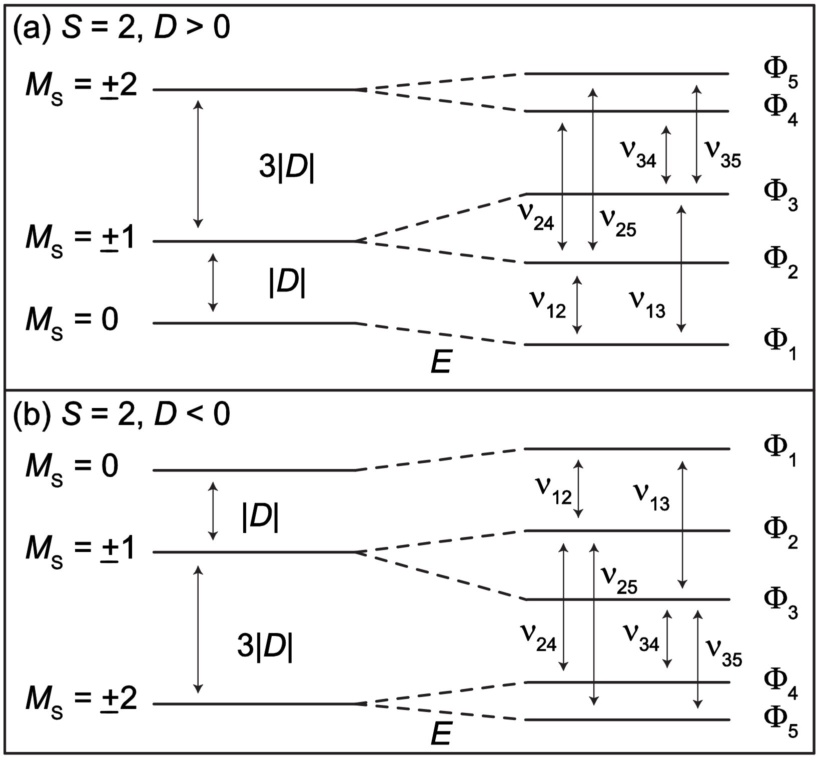

Figure S12. Magnetic sublevel energy diagrams for *S* = 2 systems with (a) positive or (b) negative *D*. With either zero *E* or nonzero *E*, the sign of *D* can be determined from the temperature-dependent spectral amplitudes.

5. Materials and pellet preparation

Microcrystalline powders of the two Co(II) compounds were synthesized according to literature procedures[10]. Anhydrous $CoCl_2$ and $CoBr_2$ were prepared by heating $CoCl_2{\cdot}6H_2O$ (Alfa Aesar) and $CoBr_2{\cdot}xH_2O$ (Alfa Aesar), respectively, to 300 °C under dynamic vacuum (100 mtorr). Triphenylphosphine (Alfa Aesar), and absolute ethanol (VWR) were used as received. Microcrystalline powders of hemin,

$[Fe(H_2O)_6](BF_4)_2$, and $NiCl_2(PPh_3)_2$ were purchased from a commercial source (Sigma Aldrich) and were used without further purification.

Approximately 200 mg of hemin and $CoX_2(PPh_3)_2$ were pressed into pellets of 13-mm diameter and ~2 mm thickness. Approximately 200 mg of $Fe(H_2O)_6(BF_4)_2$ and $NiCl_2(PPh_3)_2$ were mixed with 100 mg high-density polyethylene and the solid solutions were pressed into pellets of 13-mm diameter and ~2 mm thickness. The measurements were conducted on these pellets and the results are discussed as follows. Pellets of hemin (nominally pure powders), $CoX_2(PPh_3)_2$ (nominally pure powders) and $NiCl_2(PPh_3)_2$ (nominally pure powders mixed with HDPE) were pressed to ~2 mm thickness in air with a manual hydraulic press (MTI) using a 13 mm stainless steel pellet die (Specac) with 3 tons of applied mass. Pellets of $Fe(H_2O)_6(BF_4)_2$ (nominally pure powders mixed with HDPE) were pressed to ~2 mm thickness in a N_2-filled glovebox (Innovative Technology) with a manual hydraulic press (Specac) using a 13 mm stainless steel pellet die (Specac) with 3 tons of applied mass.

Though the diameters of the pellets were 13 mm, the focused THz beam with a spot size of approximately ~5 mm diameter throughout the 2-mm samples defined the ~40 mm^3 sample volumes that were measured. The spin number density in the samples was on the order of 10^{23} cm^{-3}. The measured signals emerged from approximately 10^{20} spins within the spot of the focused THz beam.

6. Powder X-ray diffraction

Powder X-ray diffraction (PXRD) patterns were recorded with a Bruker D8 Advance diffractometer equipped with a Göbel mirror, rotating sample stage, LynxEye detector and Cu Kα (λ = 1.5405 Å) X-ray source in a $\theta/2\theta$ Bragg-Brentano geometry. An anti-scattering incident source slit (2 mm) and an exchangeable steckblende detector slit (8 mm) were used. The tube voltage and current were 40 kV and 40 mA, respectively. Knife-edge attachments were used to remove scattering at low angles. Samples for PXRD were prepared by placing a thin layer of the designated materials on a zero-background silicon (510) crystal plate.

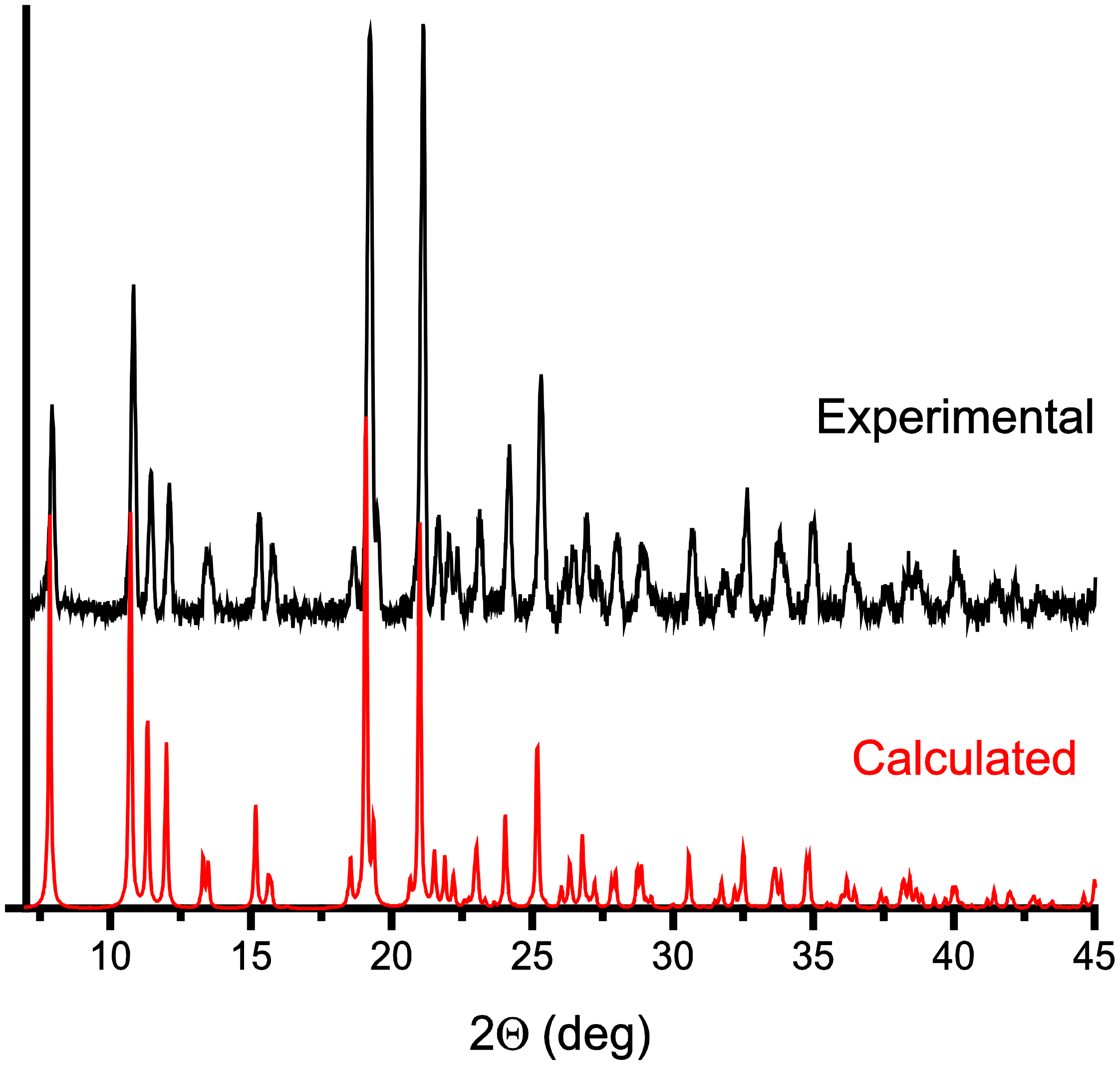

Figure S13. Background-corrected experimental (black) and calculated (red) PXRD patterns of $CoCl_2(PPh_3)_2$.

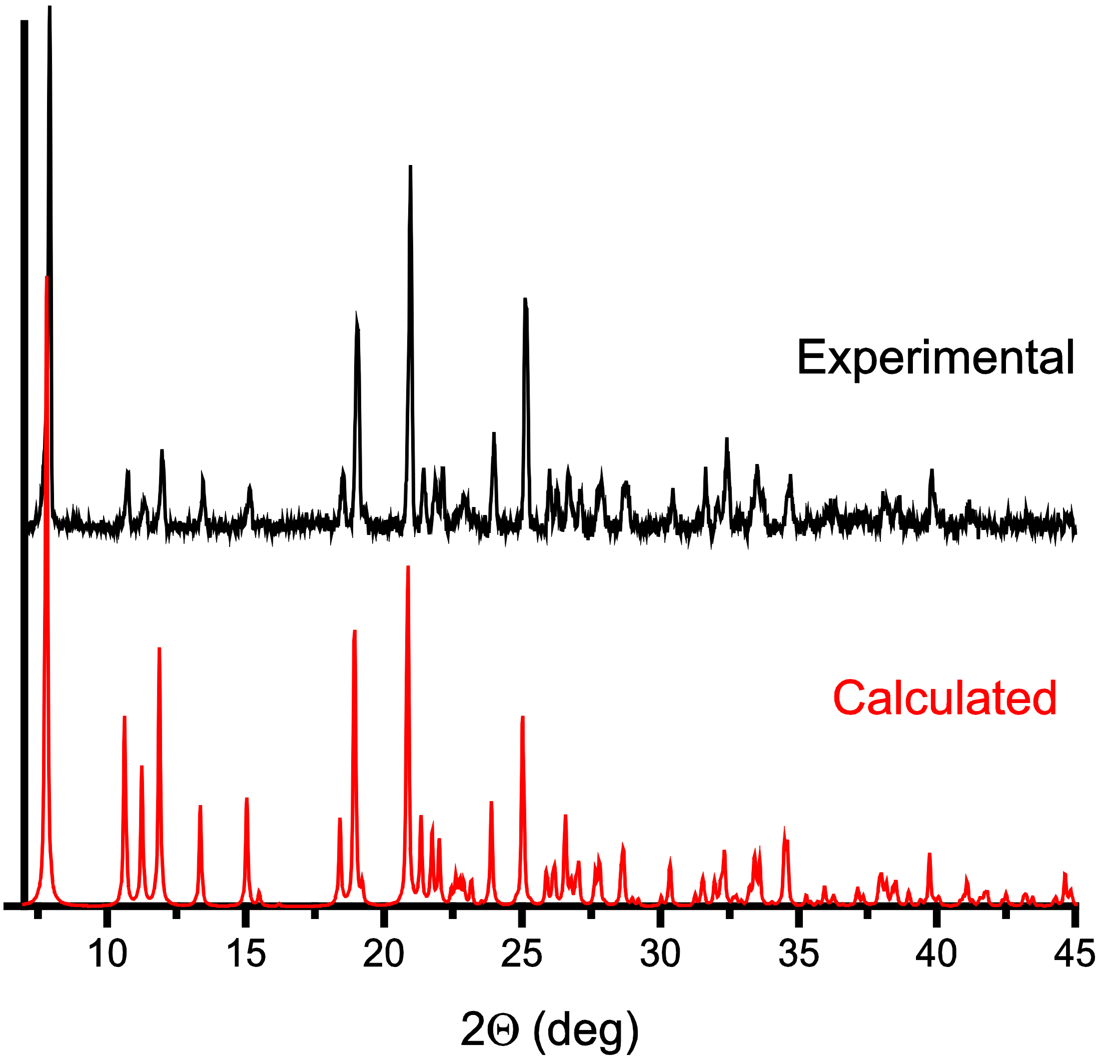

Figure S14. Background-corrected experimental (black) and calculated (red) PXRD patterns of $CoBr_2(PPh_3)_2$.

References

1 A. Nahata, A. S. Weling and T. F. Heinz, *Appl. Phys. Lett.*, 1996, **69**, 2321–2323.
2 R. Boyd, *Nonlinear optics*, Academic Press, 2007.
3 Y.-S. Lee, *Principles of terahertz science and technology*, Springer, 2009.
4 Q. Wu and X. C. Zhang, *Appl. Phys. Lett.*, 1995, **67**, 3523.
5 J. Nehrkorn, A. Schnegg, K. Holldack and S. Stoll, *Phys. Rev. Lett.*, 2015, **114**, 10801.
6 G. Hanson and L. J. Berliner, Eds., *High Resolution EPR: Applications to Metalloenzymes and Metals in Medicine*, Springer, 2009.
7 J. A. Weil and J. R. Bolton, *Electron paramagnetic resonance: elementary theory and practical applications*, Wiley, 2007.
8 J. Nehrkorn, J. Telser, K. Holldack, S. Stoll and A. Schnegg, *J. Phys. Chem. B*, 2015, **119**, 13816–13824.
9 J. Telser, J. Krzystek and A. Ozarowski, *J. Biol. Inorg. Chem.*, 2014, **19**, 297–318.
10 M. Idešicová, J. Titiš, J. Krzystek and R. Boča, *Inorg. Chem.*, 2013, **52**, 9409–9417.
